# In Vitro Study of Human Immune Responses to Hyaluronic Acid Hydrogels, Recombinant Spidroins and Human Neural Progenitor Cells of Relevance to Spinal Cord Injury Repair

**DOI:** 10.3390/cells10071713

**Published:** 2021-07-06

**Authors:** Chenhong Lin, Åsa Ekblad-Nordberg, Jakob Michaëlsson, Cecilia Götherström, Chia-Chen Hsu, Hua Ye, Jan Johansson, Anna Rising, Erik Sundström, Elisabet Åkesson

**Affiliations:** 1 Division of Neurogeriatrics, Department of Neurobiology, Care Sciences and Society, Karolinska Institutet, SE-171 64 Stockholm, Sweden; erik.sundstrom@ki.se; 2 Division of Obstetrics and Gynecology, Department of Clinical Science, Intervention and Technology, Karolinska Institutet, SE-141 52 Stockholm, Sweden; asa.ekblad@ki.se (Å.E.-N.); cecilia.gotherstrom@ki.se (C.G.); 3 Center for Infectious Medicine, Department of Medicine Huddinge, Karolinska Institutet, SE-141 86 Stockholm, Sweden; jakob.michaelsson@ki.se; 4 Department of Engineering Science, Institute of Biomedical Engineering, University of Oxford, Oxford OX3 7DQ, UK; hsuchiachen777@gmail.com (C.-C.H.); hua.ye@eng.ox.ac.uk (H.Y.); 5 Department of Biosciences and Nutrition, Karolinska Institutet, SE-141 83 Stockholm, Sweden; janne.johansson@ki.se (J.J.); anna.rising@ki.se (A.R.); 6 Department of Anatomy, Physiology and Biochemistry, Swedish University of Agricultural Sciences, SE-750 07 Uppsala, Sweden; 7 The R&D Unit, Stockholms Sjukhem, SE-112 19 Stockholm, Sweden

**Keywords:** human immune response, hyaluronic acid hydrogel, artificial spidroin, human neural progenitor cell, spinal cord injury

## Abstract

Scaffolds of recombinant spider silk protein (spidroin) and hyaluronic acid (HA) hydrogel hold promise in combination with cell therapy for spinal cord injury. However, little is known concerning the human immune response to these biomaterials and grafted human neural stem/progenitor cells (hNPCs). Here, we analyzed short- and long-term in vitro activation of immune cells in human peripheral blood mononuclear cells (hPBMCs) cultured with/without recombinant spidroins, HA hydrogels, and/or allogeneic hNPCs to assess potential host–donor interactions. Viability, proliferation and phenotype of hPBMCs were analyzed using NucleoCounter and flow cytometry. hPBMC viability was confirmed after exposure to the different biomaterials. Short-term (15 h) co-cultures of hPBMCs with spidroins, but not with HA hydrogel, resulted in a significant increase in the proportion of activated CD69^+^ CD4^+^ T cells, CD8^+^ T cells, B cells and NK cells, which likely was caused by residual endotoxins from the *Escherichia coli* expression system. The observed spidroin-induced hPBMC activation was not altered by hNPCs. It is resource-effective to evaluate human compatibility of novel biomaterials early in development of the production process to, when necessary, make alterations to minimize rejection risk. Here, we present a method to evaluate biomaterials and hPBMC compatibility in conjunction with allogeneic human cells.

## 1. Introduction

### 1.1. Spinal Cord Injury

Spinal cord injury (SCI) disrupts spinal cord function due to primary neural cell loss, hemorrhage, ischemia and secondary processes such as excitotoxicity, inflammation, demyelination and apoptosis, causing further structural damage. SCI results in vulnerability to secondary complications such as pressure ulcers, pain, urinary tract infections or progressing myelomalacia [1,2]. The local spinal environment after an injury includes multiple obstacles and little support for regeneration [3]. Multiple biomaterials have been tested to support repair, modify and enhance regenerative cues in the microenvironment, and offer drug or cell delivery for regenerative purposes after SCI [4]. Biomaterials aimed to improve structural and functional outcome after SCI require features of regenerative support while presenting low cytotoxicity and high biocompatibility, with no or minor immune reactions. 

### 1.2. Biomaterials in CNS Repair

Numerous biomaterials including different natural and synthetic polymers, derived hydrogels, acellular scaffolds and synthetic porous tubes have been evaluated for injuries and disorders in the central nervous system (CNS). Some of the most promising materials include hyaluronic acid (HA) [5,6,7], collagen [8,9], nanofibers and various 3D porous scaffolds [10,11]. Biomaterials to be applied in neuroregenerative medicine should support neural cell adhesion, migration, proliferation and differentiation. Both spider silk [12,13] and hydrogels [14,15] have been shown to be promising in these regards. Therefore, in this study, we set out to evaluate these two distinctly different but potent biomaterial subtypes regarding their human compatibility and immunogenicity. 

### 1.3. Spider Silk

Natural spider silk is a well-known high-performance fiber, which is both strong and extensible [16]. It has potential for regenerative purposes based on its original biocompatibility [17] and tolerance, as observed, for example, when implanted in vivo in a swine model [18]. In addition, Varone et al. reported no microglial reaction after implantation into rodent spinal cord [19]. Spider silk has also been shown to promote peripheral nerve injury repair in rat and sheep models by promoting Schwann cell migration, axonal regrowth and remyelination [17,20]. However, large-scale harvest as well as quality control of natural spider silk is practically difficult. 

Multiple attempts [17,20] to produce and spin recombinant spider silk have been made to mimic the potent characteristics of natural spider silk. Different types of spider silk proteins, namely spidroins, have been expressed in various heterologous host systems applying different lengths of repetitive regions and/or adding different functional domains. However, owing to large spidroin sizes with long repetitive domains and prone-to-aggregate features, the biomimetic attempts still face the challenge of low yield and low solubility [21]. We have previously presented a successful strategy to produce a recombinant miniature spidroin called NT2RepCT in a soluble form that could be spun into continuous fibers [22]. The advantages of this method are that highly soluble recombinant miniature spidroins can be produced in high yields in *Escherichia coli*. The toughness of the NT2RepCT fibers is significant, albeit lower than that of natural spider silk [22].

In order to enhance cell binding features, a cell affinity peptide, PQVTRGDVFTL from vitronectin (VN) has been added to N-terminally NT2RepCT, resulting in VN–NT2RepCT (Hansson et al. supporting manuscript). In addition, VN peptide has previously been fused to recombinant 4RepCT silk proteins, which allowed for culture of human fibroblasts and human pluripotent stem cells [23,24,25,26]. The biocompatibility of 4RepCT has previously been studied in vivo to some degree, where 4RepCT fibers were implanted subcutaneously in rats for 7 days. No obvious difference in host reaction or cell responses to the implants were observed compared to the control group without implants [27].

However, NT2RepCT and VN–NT2RepCT spidroins have so far not been evaluated concerning their human immunogenicity of relevance to their biological and clinical application.

### 1.4. Hyaluronic Acid Hydrogel

HA, or hyaluronan, is an important component of the extracellular matrix (ECM) in many tissues, including connective and epithelial tissue, as well as neuroepithelial-derived tissues of the CNS [28]. Scaffolds of naturally derived, negatively charged HA have been applied in experimental SCI. For example, high molecular weight HA was reported to decrease cell proliferation of cultured neonatal and adult astrocytes and to reduce chondroitin sulfate proteoglycan production in vitro [29]. This implied that HA based hydrogels may hold potential for minimizing undesirable scarring in SCI, which could be of significance for SCI repair strategies.

Hydrogels are three-dimensional (3D) crosslinked hydrophilic polymers of natural or synthetic origins with high water content and highly tunable mechanical properties that can be compatible with various functional molecules [30,31]. Hydrogels applied in SCI repair include a variety of naturally and synthetically derived polymers such as alginate, agarose, chitosan, collagen, fibronectin, HA, poly lactic acid (PLA) and polyethylene glycol (PEG) [32].

Mechanical properties, morphology and microarchitecture of hydrogels are all of great importance for neuroregenerative purposes. For example, stiffness-matched HA hydrogel transplanted into SCI animal models has been shown to decrease the astrocytic response significantly in the spinal cord [29]. HA hydrogel with shear modulus of roughly 1200 Pascal has also been shown to improve neuronal, especially motor neuronal, survival in mouse-derived organotypic spinal cord slice cultures and to limit microglial activation [33].

When considering the application of biomaterials in SCI repair, including neural cell therapy, degradation properties of hydrogels have also been shown to be important for maintenance of the stemness of neural progenitor cell (NPC), as well as for NPC differentiation and maturation [34,35].

HA hydrogels can be composed of different microarchitectures such as bulk or granular HA hydrogels [36]. Granular hydrogels are injectable, microporous scaffolds made from bulk hydrogels that possess self-assembling, shear-thinning and self-healing properties [37]. With the advantage of those physical properties, granular HA hydrogels have been successfully applied to culture human-induced pluripotent stem cells, hiPSCs [36]. It has also been shown that granular hydrogels may promote cellular infiltration and rather closely replicate the complex 3D structure of CNS tissues [38]. Furthermore, mechanical and biochemical properties of granular hydrogels may be fine-tuned to facilitate nutrient exchange and cell–cell interactions in the hydrogel [37].

Concerning immunological properties, HA has been reported to present low immunogenicity in in vivo rodent models [39]. However, HA may still crosstalk with the host immune system. For example, HA can bind to CD44 expressed on the cell surface of human hematopoietic cells [40]. Kajahn et al. also demonstrated that the exposure of highly sulfated HA to human monocytes, pre-induced toward M1 type macrophages in vitro, could act as an immunomodulator supporting M2 polarization [41]. The suggested mechanism behind the modulation was a disturbance of MCP-1-, IL-6- and IFN-γ-mediated M1 activation while inducing M2-related cytokine IL-10 production and CD163 expression. In addition, a significant reduction of IL-12 and TNFα levels [41] was reported. Furthermore, it was demonstrated that high molecular weight HA has intrinsic anti-inflammatory properties, presumably due to its radical oxygen species-scavenging properties [42]. Taken together, HA can potentially have immunomodulatory properties [43]. However, when it comes to the HA immunogenic potential in relation to human immune cells, it is still not fully elucidated how hydrogels with varying composition and microarchitectures interact with human lymphocytes.

Biomaterials implanted into the human body may potentially elicit an immune response through a process described as the foreign body response [44], which generally starts with an innate response with recruitment and infiltration of neutrophils and macrophages that attempt to degrade the material. In addition, these first host cell responders may trigger a release of cytokines produced by other innate immune cells [45]. Antigen presenting cells (APCs) such as macrophages and dendritic cells may thereafter present the antigens derived from biomaterials to naïve T cells, which then get activated and proliferate. The nature of the T cell response, e.g., if it is biased toward a Th1-, Th2- or Th17-type of CD4^+^ T cell response, will also dictate the subsequent response by other immune cells, and as such could affect implant integration, degradation and tissue healing. For example, Th1 CD4^+^ T cells are more prone to promote proinflammatory M1 macrophage activation, Th2 CD4^+^ T cells are more prone to promote more anti-inflammatory M2 macrophages, and Th17 CD4^+^ T cells can induce recruitment and activation of neutrophils [46]. Biomaterials implanted in the CNS may cause a chronic inflammation with fibrotic lesion formation similar to a scar after a CNS injury to restrict the injury site [47]. Physical properties of the biomaterials, like shape, surface features and electrical charge, have been shown to affect the immunological response, as described by Andorko et al. and O’Shea et al. [46,47]. In regenerative medicine, a certain degree of a controlled immune response may even be desired to slowly degrade an implanted biomaterial scaffold while supporting the transplanted cells to survive and proliferate until full integration into the host tissue is achieved.

Taken together, previous and present research indicate that both HA hydrogels and recombinant spidroins are interesting components that may serve as potent base substrates in human SCI repair. In order to develop treatment strategies and apply these biomaterials as part of composite approaches for SCI, it is of value to investigate their biocompatibility and immunogenicity in clinically relevant human cell cultures. Herein, we present an in vitro study observing how human peripheral blood mononuclear cells, hPBMCs, reacted to recombinant miniature spidroins (either the NT2RepCT or VN–NT2RepCT protein, in the form of film and soluble protein) or HA hydrogels (either in its bulk or granular form). We also co-cultured hPBMCs with allogeneic human spinal cord derived NPCs (hNPCs) in the presence of these biomaterials, aiming to mimic an allogeneic host–donor interaction when applying biomaterial as part of a composite experimental intervention. We monitored the human immune cell viability, proliferative response and evaluated different immune cell phenotypes after co-culture using NucleoCounter and flow cytometry.

## 2. Materials and Methods

### 2.1. Ethical Considerations

Human neural cells were collected after oral and written informed consent following ethical permission from the Regional Human Ethics Committee, Stockholm/Swedish Ethical Review Authority (Dnr 2007/1477-31, 2011/1101-32, 2013/654-32 and 2018/2497-32) and The National Board of Health and Welfare (Dnr 8.1-544/2018) as well as in accordance with the Declaration of Helsinki.

### 2.2. Isolation and Culture of Human Spinal Cord Derived Neural Progenitor Cells

Human 1st trimester residual tissues, including pieces of embryonic/fetal spinal cord tissue (5.5–9.5 postconceptional weeks, n = 3) were collected after legal elective abortions and informed consent according to ethical permissions. Dissections were performed under sterile conditions under a dissection microscope. The spinal cord tissue was identified, staged and rinsed in sterile sodium chloride at physiological concentration. hNPCs were generated, characterized and propagated as previously described [48,49]. Briefly, the spinal cord tissue was mechanically homogenized with a Teflon–glass homogenizer. The obtained cell suspension was seeded in non-tissue culture treated flasks as free-floating neurospheres at a density of 1 × 10^5^–1.5 × 10^5^ cells/mL in the hNPC medium (DMEM:F12; 1:1 (Life Technologies, Carlsbad, CA, USA) supplemented with 0.6% glucose, 5 mM N-2-hydroxyethylpiperazine-N-2-ethane sulfonic acid (HEPES, Gibco, Life Technologies), 2 μg/mL heparin (Sigma-Aldrich, St. Louis, MO, USA), 1% N-2 supplement (Life Technologies), 20 ng/mL recombinant epidermal growth factor (EGF, R&D Systems, Minneapolis, MN, USA), 20 ng/mL basic fibroblast growth factor (bFGF, R&D systems) and 10 ng/mL ciliary neurotrophic factor (CNTF, R&D Systems)). 

The cells were maintained in humidified 5% CO_2_ at 37 °C. Fresh medium was added twice per week. The neurospheres were passaged every 14–25 d (depending on the growth of the individual culture) with TrypLE Express Enzyme (1X, no phenol red, Gibco, Life Technologies) treatment and thereafter resuspended and cultured in fresh medium. The characteristics of the hNPCs applied here was confirmed by immunohistochemistry to present similar features as previously reported [48,50].

### 2.3. Artificial Spidroins

The recombinant miniature spidroins NT2RepCT and VN–NT2RepCT were produced in *E. coli* and purified as previously described [22].

To coat the cell culture plate with spidroin film, the coating solution was prepared by mixing the following in the order as shown: 20 mM TrisHCL pH 8, 100 µg/mL NT2RepCT or VN–NT2RepCT dissolved in 20 mM TRIS, 20 mM HEPES/Mes, and 2.5 mg/mL glucono–delta–lactone (GDL). Then, 24-well cell culture plates (non-tissue culture treated surface, flat bottom, polystyrene, Nunc, Thermo Scientific, Waltham, MA, USA) were filled with 150 µL of the respective coating solution per well for 5 min. Thereafter the solution was removed, and the plates were incubated overnight in 37 °C prior to use.

For adding soluble spidroins in the cultures, the soluble NT2RepCT or VN–NT2RepCT were directly added to the hPBMC cultures to a final concentration of 100 µg/mL. In a prior control experiment, different spidroin protein concentrations ranging from 0–300 µg/mL were tested and confirmed to not affect hPBMC viability. For details, see below and Figure S1.

### 2.4. Bulk and Granular HA Hydrogel Fabrication

The bulk and granular HA hydrogels were prepared according to a method developed by Prof. Hua Ye’s lab [51], using HyStem^®^ cell culture scaffold kit (Sigma-Aldrich) to fabricate HA hydrogels following a modified version of the manufacturer’s recommended protocols.

The dehydrated hyaluronan/PBS (HyStem) was reconstituted with 500 µL of degassed water making a 2 *w*/*v*% HyStem solution. The crosslinker from the kit (Extralink^®^-1) was reconstituted with 250 µL degassed water, making a 2 *w*/*v* % Extralink^®^-1 solution.

For bulk hydrogels, 1:1 (*v/v*) ratio of NS medium (DMEM:F12; 1:1 (Life Technologies) supplemented with glucose (0.6%, Sigma-Aldrich), HEPES (5 mM, Gibco, Life Technologies), heparin (2 μg/mL, Sigma-Aldrich) and N-2 supplement (1%, Life Technologies)), together with 125 µL of the 2 *w*/*v*% Extralink-1, were added to the 500 µL 2 *w*/*v*% HyStem solution, resulting in 1250 µL of 1 *w*/*v*% bulk HA hydrogel after gelling at 37 °C for 30 min. 

For granular hydrogels, 125 µL of the 2 *w*/*v*% Extralink-1 was mixed with 500 µL 2 *w*/*v*% HyStem solution, and the system was pre-gelled at 37 °C for 2.5 h. Once gelled, hydrogel was granularized by extruding from 1 mL syringe through the gel fragment channel of a three-way combined device with an 8 mm nylon mesh disc with pore size of 40 µm in diameter. A 1:1 (*v/v*) ratio of PBS/medium and the secondary crosslinker Extralink-1 with the concentrations of 20 *v/v*% primary crosslinker was added to the gel fragments and mixed. The mixed system was gelled at 37 °C for 30 min to form a final volume of 1 *w*/*v*% granular HA hydrogel. 

### 2.5. hNPC Encapsulation in Bulk and Granular HA Hydrogels

For cellular encapsulation in granular HA hydrogels, 2 *w*/*v*% HyStem hydrogel fragments were fabricated as described in the above section using the autoclaved three-way combined device. First, 2 × 10^6^ cells/mL of hNPCs were resuspended in NS medium supplemented with 200 µg/mL laminin and 50 ng/mL brain-derived neurotrophic factor (BDNF). A total of 400 µL of the hydrogel fragments were then mixed with an equal volume of the hNPC suspension, together with 16 µL of 2 *w*/*v*% secondary Extralink-1. The final 1 *w*/*v*% hNPC encapsulated granular HA hydrogel composites with ~10^6^ cells/mL hNPCs were incubated and gelled at 37 °C for 30 min after the mixture.

For bulk hydrogels, 400 µL of 2 *w*/*v*% uncrosslinked HyStem solution was mixed with an equal volume of the hNPC suspension, together with the same amount of total Extralink-1 added into the granular HA hydrogel composites, using a 1 mL syringe, followed by gelation at 37 °C for 30 min after the mixture, making a final bulk HA hydrogel of ~10^6^ cells/mL hNPCs.

After hydrogel formation, the bulk and granular HA hydrogels were cast as spheroids with a volume of 50–60 µL/spheroid. The hydrogel spheroid was gently transferred into 24-well cell culture plates (flat bottom TPP^®^ tissue culture plates, Sigma-Aldrich), topped up with NS medium supplemented with 10 ng/mL BDNF to support neuronal differentiation, and maintained by half media exchange every 3 d.

### 2.6. hPBMC Preparation

hPBMCs were isolated from buffy coats collected from healthy donors (n = 5) by density gradient separation (Lymphoprep; Nycomed Pharma, Zürich, Switzerland) at 500 *g* for 20 min at room temperature. The hPBMCs were either used directly after isolation or cryopreserved before use. At the day of culture, the hPBMCs were seeded in 24-well cell culture plates at a concentration of 1 × 10^6^/mL in RPMI 1640 medium (Life Technologies) supplemented with 10% pooled inactivated human AB serum, 100 U/mL penicillin, 100 mg/mL streptomycin (Gibco, Life technology), 20 U/mL recombinant IL-2 (R&D Systems) and 20 mM L-glutamine (Invitrogen, Waltham, MA, USA). Prior to each culture, a cell count and viability test were performed on all the hPBMCs, as described below. Some of the hPBMCs were stimulated with T Cell Activation/Expansion Kit (human, Miltenyi Biotec, Bergisch Gladbach, North Rhine-Westphalia, Germany) to promote activation and proliferation of T cell subsets in the hPBMCs, which method will be abbreviated to TCA in the rest of the text. Briefly, Anti-Biotin MACSiBead™ particles were loaded with biotinylated CD2, CD3 and CD28 antibodies and incubated for 2 h at 2–8 °C under gentle rotation. The loaded beads were washed and mixed with the hPBMCs in a ratio of 1 × 10^6^ beads per 2 × 10^6^ hPBMCs.

### 2.7. Viability Test and Cell Count

As a first step, hPBMC tolerance toward the two different biomaterials (in different forms) were tested. Prior to analysis, we had determined the spidroin concentration to be applied by having hPBMC exposure for 5–6 h, 24 h or 4 d to soluble NT2RepCT or VN–NT2RepCT of different concentrations (1 µg/mL, 10 µg/mL, 100 µg/mL or 300 µg/mL) and measured hPBMC viability, as illustrated in Figure S1.

In the subsequent proliferation assay, a spidroin protein concentration of 100 µg/mL was applied, since it resulted in an unchanged viability compared to no spidroin protein added, as well as was the equivalent concentration applied as when preparing spidroin film. A concentration of 100 µg/mL of soluble spidroin, as applied here, is relatively high, considering that future applications will include implantation of spidroin fiber or film but not spidroin solution. Exposure of soluble spider silk form to the host immune system will, therefore, only be expected to occur after biomaterial degradation takes place over time in the host after implantation.

Human PBMCs were cultured for 5–6 h, 24 h and 4 d with bulk HA hydrogel (≈50 µL/piece in each well), granular HA hydrogel (≈50 µL/piece in each well) and soluble or film of NT2RepCT or VN–NT2RepCT (100 µg/mL). Thereafter, the cells were collected and stained with Acridine Orange (to stain both live and dead cells, ChemoMetec, Allerød, Hovedstaden, Denmark) and with DAPI (4’,6-diamidino-2-phenylindole, to detect live cells, ChemoMetec). Viability and total cell count of the hPBMCs were thereafter evaluated in a NucleoCounter (NC-3000, ChemoMetec) according to the manufacturer’s instruction.

### 2.8. hPBMC Response to Biomaterials and hNPCs

To investigate whether the two different types of biomaterials or the hNPCs induced an immune response, 1 × 10^6^ hPBMCs were co-cultured with allogeneic 1 × 10^5^ hNPCs and/or biomaterials and thereafter analyzed.

#### 2.8.1. Short-Term (15–18 h) Co-Culture and Subpopulation Activation Assay

To evaluate the immediate hPBMC activation level, freshly isolated hPBMCs were seeded into 24-well flat-bottom plates under the following conditions: (1) hPBMC, only (control); (2) hPBMCs + lipopolysaccharide (LPS control, 10 ng/mL, Sigma-Aldrich); (3) hPBMCs + T cell activation (TCA control, Miltenyi Biotec); (4) hPBMCs + VN–NT2RepCT film; (5) hPBMCs + granular HA hydrogel (50 µL/piece in each well; illustrated as “G-gel” in figures); (6) hPBMCs + hNPCs; (7) hPBMCs + VN–NT2RepCT film + hNPCs; and (8) hPBMCs + granular HA hydrogel with hNPCs encapsulated (illustrated as “G-gel + hNPCs” in the figures). The density of the hPBMCs were 1 × 10^6^ per well, while the density of the hNPCs was 1 × 10^5^ per well. The cells were kept in culture for 15–18 h. Thereafter, the cells were pelleted by centrifugation at 300× *g* for 5 min, followed by immunolabeling in 96-well plates with 50 µL of an antibody cocktail (as described in Table S1) for 30 min at 4 °C. All antibodies were diluted in PBS with 2% fetal bovine serum (FBS) and 2 mM EDTA (FACS-wash).

The activation of T cells, B cells and NK cells was assessed here by analyzing CD69 surface molecules, which is a classical early activation marker of lymphocytes [52]. For T cells, another activation marker, 4-1BB, was also evaluated, which is not only induced upon T cell activation but also remains on activated T cells [53].

#### 2.8.2. Long-Term (5 d) Co-Culture and Proliferation Assay

In order to further evaluate the hPBMCs immunoreaction to the two different biomaterials, 1 × 10^6^ stimulated or resting hPBMCs were seeded per well in 24-well plates under the following conditions and cultured for 5 days: (1) hPBMC, only; (2) hPBMCs + soluble NT2RepCT; (3) hPBMCs + NT2RepCT film; (4) hPBMCs + soluble VN–NT2RepCT (“VN–NT2RepCT sol.”); (5) hPBMCs + VN–NT2RepCT film; (6) hPBMCs + bulk HA hydrogel (illustrated as “B-gel” in the figures); (7) hPBMCs + bulk HA hydrogel with hNPCs encapsulated (illustrated as “B-gel + hNPCs” in figures); (8) hPBMCs + granular HA hydrogel; and (9) hPBMCs + granular HA hydrogel with hNPCs encapsulated.

Prior to the seeding of hNPCs, neurospheres were digested as described above and passed through a cell strainer (40 µm, BD Falcon, BD Bioscience, Franklin Lakes, NJ, USA) to obtain single cells.

At day 0, frozen hPBMCs were thawed and stained with 10 μM CFSE (carboxyfluorescein succinimidyl ester) (CellTrace™ Cell Proliferation Kit, Life Technologies) according to the manufacturer’s instructions. hPBMCs were resuspended in PBS at 10 × 10^6^ cells/mL; CFSE stock solution was added to a final concentration of 10 μM CFSE. The hPBMCs were incubated in 37 °C for 10 min. Thereafter, the staining was quenched by addition of 3 mL FBS per mL cell suspension and incubated for another 5 min at room temperature. The PBMCs were then washed and resuspended in regular culture medium as described above. The fluorescent intensity level of CFSE was evaluated with flow cytometry in order to determine the proliferation level of the hPBMCs.

To analyze the cell proliferation, hPBMCs were harvested at day 0 (D0) and day 5 (D5). The cells were stained with LIVE/DEAD™ Fixable Aqua Dead Cell Stain Kit (Invitrogen) to determine the viability of cells and with antihuman CD3-APC for T cell detection (5 µL/1 × 10^6^ cells, BD Biosciences), according to manufacturer’s instruction. Thereafter the cells were fixed with BD Cytofix/Cytoperm kit (BD Pharmingen, BD Bioscience).

The cell proliferation was evaluated by analyzing the fluorescence intensity of CFSE by flow cytometry. The CFSE^low^ population included the highly proliferated population (the CFSE cellular content diluted), while the CFSE^high^ included the non-proliferated population.

### 2.9. Flow Cytometry, Gating Strategies and Data Analysis

The flow cytometry data from the activation assay as described above under “short-term co-culture” was acquired on a FACSymphony SORP instrument (BD Biosciences), while analysis of the proliferation assay after the “long-term co-culture” was acquired on a FACS Verse (BD Biosciences). All flow cytometry data was analyzed with FlowJo software (v.10, Tree Star, Ashland, OR, USA). The applied gating strategies for the activation assay data and proliferation assay data are illustrated, respectively, in Figures S2 and S3, where arrows were applied in the figure legend to illustrate successive order of gating.

### 2.10. Limolus Amebocyte Lyste Test

A limolus amebocyte lyste (LAL) test (Pierce^TM^ LAL Chromogenic Endotoxin Quantitation test. No. 88282, Thermo Scientific) of NT2RepCT and VN–NT2RepCT proteins in solution was carried out according to the manufacturer’s instructions. NT2RepCT was tested at a concentration of 3 mg/mL and VN–NT2RepCT at 2.6 mg/mL and serial dilutions thereof (10×, 100× and 1000× in endotoxin free H_2_O), as illustrated in Figure S4.

### 2.11. Statistics

GraphPad Prism 8.0 (GraphPad Software, La Jolla, CA, USA) was used for statistical analysis and production of graphical illustration.

The normally distributed parametric paired data from the viability assay was analyzed with repeated measure one-way analysis of variance (ANOVA) with Dunnett’s test as post hoc test to compare the mean of the multiple experimental groups with the mean of the control group (hPBMC, only).

The normally distributed paired data with unequal sample sizes from the activation assay was analyzed with mixed-effects model ANOVA (with Geisser–Greenhouse correction). When a significant difference was observed, the Dunnett’s test was applied as post hoc test to compare the experimental groups with the control groups (hPBMC-only group or TCA group), while reporting multiplicity adjusted p value for each comparison.

The paired data with unequal sample sizes from the proliferation assay was first log-transformed then analyzed with mixed-effects analysis with Geisser–Greenhouse correction. When a significant difference was observed in the omnibus test, the Dunnett’s test was applied as post hoc test to compare the experimental groups with the control groups (hPBMC-only group or TCA group), while reporting multiplicity adjusted p value for each comparison. *p*-values ≤ 0.05 were considered statistically significant.

Data are presented with mean and standard deviation (SD) in the corresponding graphs.

## 3. Results

The different biomaterials (film/soluble NT2RepCT and VN–NT2RepCT, bulk/granular HA hydrogels with/without hNPCs) were prepared, co-cultured and evaluated concerning their influence on hPBMCs, with respect to cytotoxicity, activation of different subsets of lymphocytes and proliferation of human T- and non-T cell populations.

### 3.1. Spidroins or HA Hydrogel Did Not Compromise the Viability of hPBMCs

**Figure 1:**
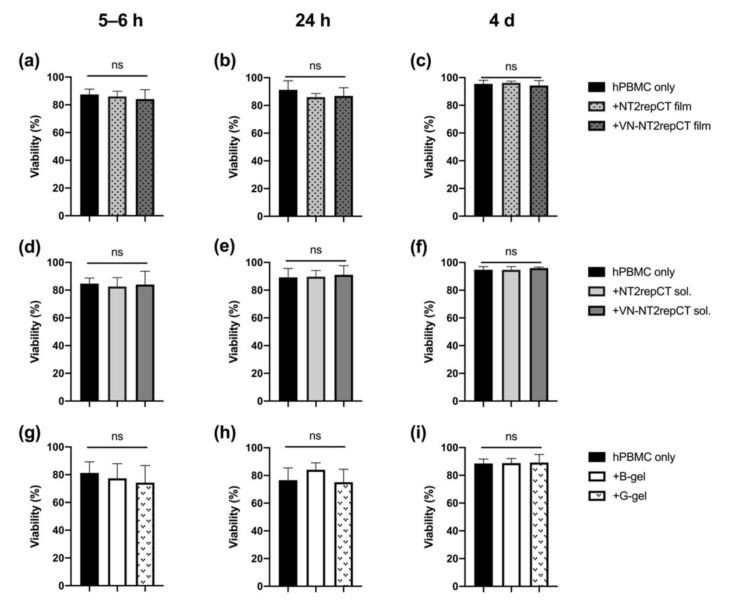
Viability of hPBMCs after exposure to the biomaterials. The hPBMCs were kept in culture with (**a**–**c**) NT2RepCT and VN–NT2RepCT film, (**d**–**f**) soluble NT2RepCT and VN–NT2RepCT and (**g**–**i**) bulk (“B-gel”) and granular (“G-gel”) HA hydrogel for 5–6 h, 24 h and 4 d, and thereafter, the cellular viability was measured. Data are presented as mean + SD. The statistical test repeated measure ANOVA was applied to compare the mean of the experimental groups with the mean of the control group (hPBMC, only). ns: not significant, *p* > 0.05. n = 4 biologically different hPBMC populations. Abbreviations: SD, standard deviation; sol., soluble; B-gel, bulk HA hydrogel; G-gel, granular HA hydrogel.

In order to evaluate if any of the biomaterials applied were cytotoxic to the immune cells, the hPBMCs were exposed to the respective biomaterials. The hPMBC viability was measured at 5–6 h, 24 h and 4 d after cell seeding and initial exposure. The viability of the hPBMCs was consistently high, with no significant difference in the viability, with or without the presence of the biomaterials selected (Figure 1, *p* > 0.05 for all groups).

### 3.2. Spidroin, but Not HA Hydrogel, Activated Immune Cells In Vitro

To analyze whether the two different types of biomaterials activated immune cells among the hPBMC populations, we co-cultured hPBMCs with VN–NT2RepCT film or granular HA hydrogel for 15–18 h. hPBMCs in culture without treatment were used as baseline control. hPBMCs with addition of LPS or anti-CD2/CD3/CD28-coated beads (TCA) were included as positive controls. Immune cell activation was analyzed by detection of cell surface expression of the activation markers CD69 and 4-1BB on T cells, B cells and NK cells using flow cytometry. The gating strategy to identify the different cell types is shown in Figure S2. Briefly, CD4^+^ and CD8^+^ T cells were identified as live CD14^−^CD15^−^CD3^+^ cells expressing either CD4 or CD8 (Figure S2). B cells were identified as live CD14^−^CD15^−^CD3^−^CD56^−^CD19^+^ cells, and NK cells as live CD14^−^CD15^−^CD3^−^HLA-DR^−^CD56^+^CD16^+^ cells.

**Figure 2:**
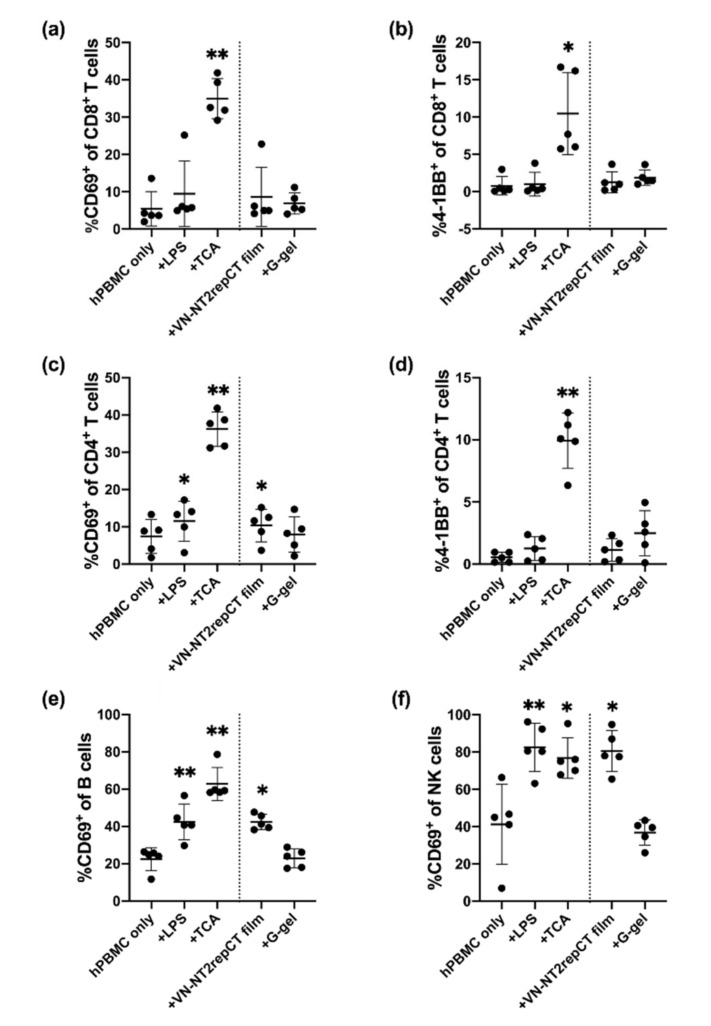
Activation of hPBMC lymphocyte subsets after being exposed to different biomaterials. hPBMCs were cultured for 15–18 h as hPBMC, only (baseline control); (hPBMCs) + LPS (positive control); (hPBMCs) + TCA (positive control); (hPBMCs) + VN–NT2RepCT film; and (hPBMCs) + Granular HA hydrogel (“G-gel”). Thereafter, the expression of CD8, CD4, CD69 and 4-1BB was assessed by flow cytometry. The graphs show the percentages of (**a**) CD69^+^ out of all CD8^+^ T cells; (**b**) 4-1BB^+^ out of all CD8^+^ T cells; (**c**) CD69^+^ out of all CD4^+^ T cells; (**d**) 4-1BB^+^ out of all CD4^+^ T cells; (**e**) CD69^+^ out of all B-cells; and (**f**) CD69^+^ out of all NK cells. The gating for B cells (CD3^−^CD56^−^CD19^+^), NK cells (CD3^−^CD56^+^CD16^+^), CD4^+^ T cells (CD3^+^CD4^+^) and CD8^+^ T cells (CD3^+^CD8^+^) is described in Figure S2. Data are presented as mean ± SD, and each dot within a group represents hPBMCs from one separate biological donor. The vertical dotted line in the graph demarcates the control versus the experimental groups. The statistical test of mixed-effects analysis with Dunnett’s post hoc test was applied to compare the experimental groups with the hPBMC-only control. Statistically significant differences from the hPBMCs-only control are indicated as: * *p* ≤ 0.05, ** *p* ≤ 0.01. n = 5 biologically different hPBMC populations. Abbreviations: SD, standard deviation; TCA, T cell activation; G-gel, granular HA hydrogel.

Stimulation with TCA resulted in a more than three-fold increase in the proportion of activated (CD69^+^) CD8^+^ T cells (35.0% ± 5.36%, Figure 2a) and CD4^+^ T cells (36.2% ± 4.61%, Figure 2c) compared to the hPBMC-only control (5.44% ± 4.61%, Figure 2a; 7.41% ± 4.55%, Figure 2c) as well as the LPS control (9.47% ± 8.81%, Figure 2a; 11.5% ± 5.38%, Figure 2c, *p* ≤ 0.05). This shows that the T cell subpopulation of the hPBMCs under the present conditions was functional and could be triggered to respond strongly.

Interestingly, the proportion of CD69^+^ cells out of the total CD4^+^ T cell population was significantly increased (10.3% ± 4.38%, *p* = 0.0120) after exposure to VN–NT2RepCT film, similar to the LPS (11.5% ± 5.38%, *p* = 0.0165) and TCA (36.2%% ± 4.61%, p = 0.0037) positive controls when compared to the hPBMC-only control group (7.41% ± 4.55%, Figure 2c). In addition, the proportion of CD69^+^ B-cells (42.4% ± 4.04%, *p* = 0.0174) and CD69^+^ NK cells (80.6% ± 11.0%, *p* = 0.0104) was significantly increased in the VN–NT2RepCT film group compared to the control with hPBMC, only, while we found no upregulation in the granular HA hydrogel group applying the same markers and conditions (Figure 2c,e,f).

There was no statistically significant difference in the proportion of activated T cells (4-1BB^+^) in the CD8^+^ T cell population after exposure to VN–NT2RepCT film (1.28% ± 1.41%, *p* = 0.0608) (Figure 2b) nor in the CD4^+^ T cell population (1.14% ± 0.899%, *p* = 0.2549) (Figure 2d) compared to the hPBMC-only group. There was also no significant difference in the 4-1BB^+^ CD8^+^ (1.89% ± 1.03%, *p* = 0.5309) nor CD4^+^ T cell populations (2.49% ± 1.81%, *p* = 0.1460) in the presence of granular HA hydrogels when compared with the baseline hPBMC-only control (*p* > 0.05) (Figure 2b,d).

### 3.3. Soluble Spidroins Stimulated the Proliferation of hPBMCs In Vitro

**Figure 3:**
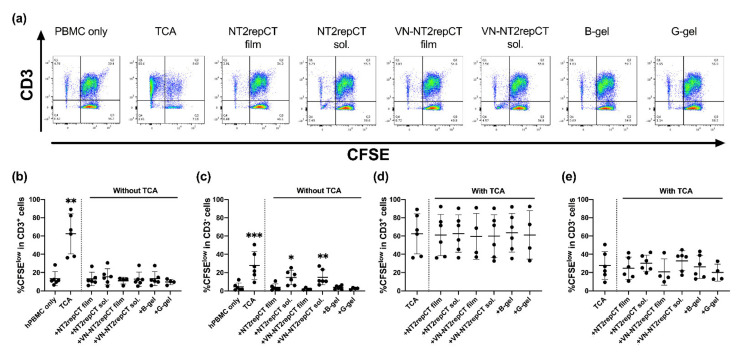
Proliferation of hPBMCs after exposure to different biomaterials for 5 d. (**a**) Representative flow cytometry data of the hPBMCs CFSE fluorescence intensity and frequency of CD3 expression. Percentages (%) of proliferated CFSE^low^ cells out of the total number of (**b**) CD3 ^+^ cells or (**c**) CD3^−^ cells in the groups, including: hPBMC, only (baseline control); (hPBMCs) + TCA (positive control); (hPBMCs) + NT2RepCT film; (hPBMCs) + soluble NT2RepCT; (hPBMCs) + VN–NT2RepCT film; (hPBMCs) + soluble VN–NT2RepCT; (hPBMCs) + bulk HA hydrogel (“B-gel”) or (hPBMCs) + granular HA hydrogel (“G-gel”) were examined. The percentages (%) of proliferated CFSE^low^ cells among the total number of (**d**) CD3^+^ cells or (**e**) CD3^−^ cells after TCA treatment in all the experimental groups was also examined. Data are presented as mean ± SD. The vertical dotted line in the graph demarcates the control versus the experimental groups. The statistical test of mixed-effects analysis with Dunnett’s post hoc test was applied after log-transformation in order to compare the experimental groups with the respective control group. Statistically significant differences from the control hPBMCs-only group (**b**,**c**) or from the TCA group (**d,e**) are indicated as: * *p* ≤ 0.05, ** *p* ≤ 0.01, *** *p* ≤ 0.001. n = 4–6 biologically different hPBMC populations. Abbreviations: SD, standard deviation; TCA, T cell activation; sol., soluble; B-gel, bulk HA hydrogel; G-gel, granular HA hydrogel.

To analyze the long-term (5d) responses of hPBMCs to different forms of the respective biomaterial in vitro, the proliferation of CD3^+^ and CD3^−^ lymphocytes was assessed subsequent to the evaluation of lymphocyte activation. The hPBMCs were cultured with or without either soluble or film form of NT2RepCT and VN–NT2RepCT or bulk or granular HA hydrogels. As positive controls, the hPBMCs were cultured with an addition of either LPS or TCA. After 5 d in culture, the fluorescence intensity of CFSE in the cell populations was analyzed by flow cytometry, as illustrated in the representative FACS plots (Figure 3a). The CFSE^low^ population included cells that had proliferated extensively.

None of the biomaterials triggered an increase in the proportion of CFSE^low^ proliferating CD3^+^ T cells compared to the hPBMC-only control group (Figure 3b, *p* > 0.05). Only the positive control, hPBMCs with TCA treatment (62.4% ± 22.0%, *p* = 0.0046), resulted in an increase (close to sixfold) in the CFSE^low^ CD3^+^ T cell subpopulation compared to the control hPBMC-only group (13.2% ± 7.69%, Figure 3b).

As for the CD3^−^ cells, the proportion of the CFSE^low^ populations were significantly increased after exposure both to the soluble NT2RepCT (14.6% ± 7.90%, *p* = 0.0256) and the film VN–NT2RepCT groups (14.9% ± 8.27%, *p* = 0.0016) compared to the hPBMC-only control (4.36% ± 4.20%) (Figure 3c).

### 3.4. No Observed Biomaterial Immunomodulatory Properties

In order to assess potential immunomodulatory properties of the biomaterials, TCA-stimulated hPBMCs were cultured with and without the biomaterials for 5 days. There was no significant increase or decrease in the CFSE^low^ CD3^+^ or CD3^−^ populations of the hPBMCs cultured with any biomaterial compared to the TCA only group (control) (Figure 3d,e, *p* > 0.05).

### 3.5. Evaluation of hPBMC Activation in Co-Culture with hNPCs, with or without Presence of Biomaterials

The biomaterials we studied are potential candidates as cell transplantation scaffolds for repair of spinal cord injury. Therefore, the two different biomaterials were also applied together with allogeneic hNPCs in hPBMC co-cultures to examine the combinatorial effects of both the transplanted cells and biomaterials.

Human PBMCs were seeded with cell suspensions of hNPCs (from dissociated neurospheres), alone, with VN–NT2RepCT film placed on the bottom of the wells of the culture plates or with hNPCs encapsulated in granular HA hydrogel for 15–18 h prior to flow cytometric analysis.

**Figure 4:**
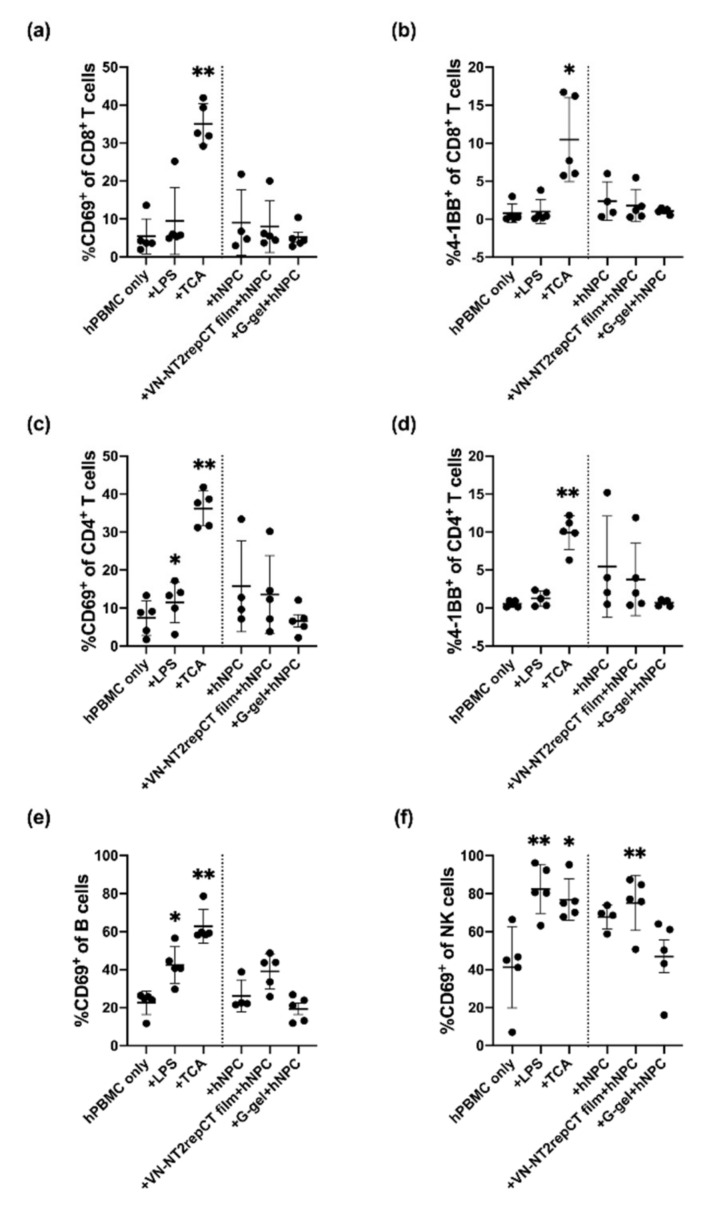
Activation of hPBMC subpopulations after co-culture with allogeneic NPCs with or without the presence of VN–NT2RepCT film or granular HA hydrogel. Human PBMCs were cultured as: hPBMC-only (baseline control); (hPBMCs) + LPS (positive control); (hPBMCs) + TCA (positive control); (hPBMCs) + cell suspension of hNPCs (“hNPCs”); (hPBMCs) + cell suspension of hNPCs with VN–NT2RepCT film (“VN–NT2RepCT film + hNPCs”) or (hPBMCs) + granular HA hydrogel with hNPCs encapsulated. After 15–18 h in culture, the expression of the activation markers CD69 and 4-1BB was analyzed by flow cytometry. The percentages (%) of hPBMC subpopulations (**a**) of CD69^+^ out of all CD8^+^ T cells; (**b**) of 4-1BB^+^ out of all CD8^+^ T cells; (**c**) of CD69^+^ out of all CD4^+^ T cells; (**d**) of 4-1BB^+^ out of all CD4^+^ T cells; (**e**) of CD69^+^ out of all B-cells; and (**f**) of CD69^+^ out of all NK cells. Data are presented as mean ± SD, and each dot within a group represents hPBMCs from one biological donor. The vertical dotted line in the graph demarcates the control versus the experimental groups. The statistical test of mixed-effects analysis with Dunnett’s post hoc test was applied to compare the experimental groups with the hPBMC-only group. Statistically significant differences from the hPBMCs-only group are indicated as: * *p* ≤ 0.05, ** *p* ≤ 0.01. n = 5 biologically different hPBMC populations. Abbreviations: SD, standard deviation; TCA, T cell activation; G-gel, granular HA hydrogel.

hNPCs, alone, did not significantly increase the proportion of activated CD69^+^ nor 4-1BB^+^ cells in any lymphocyte subpopulation compared to the hPBMC-only control group (Figure 4, *p* > 0.05).

However, when cell suspensions of hNPCs were co-cultured with VN–NT2RepCT film, the proportion of CD69^+^ NK cells (75.2% ± 14.5%, *p* = 0.0032) increased significantly compared to the hPBMC-only group (41.3% ± 21.5%). In contrast, the granular HA hydrogel encapsulating hNPCs did not cause any significant change when applying any of the activation markers of hPBMC subpopulations in the co-cultures compared to the hPBMC-only control group (Figure 4, *p* > 0.05).

### 3.6. Evaluation of hPBMC Proliferation in Co-Culture with Allogeneic NPCs with or without the Presence of Two Different Forms of Hydrogel

**Figure 5:**
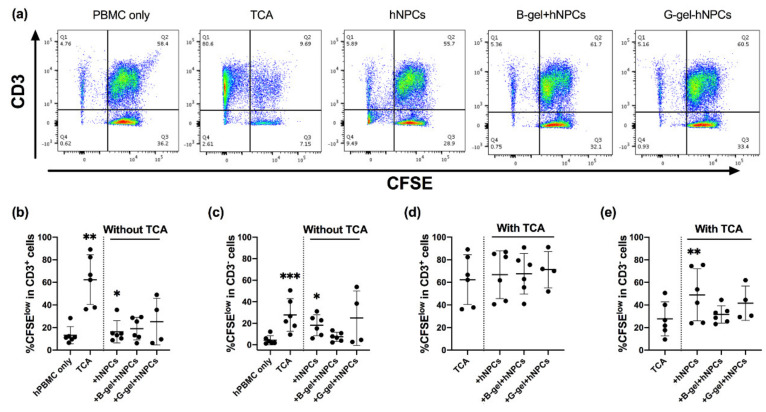
Proliferation of hPBMC subpopulations after co-culture with hNPCs in cell suspension or encapsulated in bulk or granular HA hydrogels for 5 d. (**a**) Representative flow cytometric data of the CFSE fluorescence intensity and frequency of CD3 expression. Frequencies of proliferated cells among resting (**b**) CD3 ^+^ cells and (**c**) CD3^−^ cells cultured with cell suspension of hNPCs or bulk/granular HA hydrogels with hNPCs encapsulated (with hPBMC-only groups and TCA-only groups as controls) or cultured in the same conditions after TCA treatment (**d**,**e**). Data are presented as mean ± SD. The vertical dotted line in the graph demarcates the control versus the experimental groups. The statistical test of mixed-effects analysis with Dunnett’s post hoc test was applied after log-transformation in order to compare the experimental groups with the control groups. Statistically significant differences from the hPBMCs-only group (**b**,**c**) or from the TCA group (**d,e**) are indicated as: * *p* ≤ 0.05, ** *p* ≤ 0.01, *** *p* ≤ 0.001. n = 4 biologically different hPBMC populations. Abbreviations: TCA, T cell activation; B-gel, bulk HA hydrogel; G-gel, granular HA hydrogel.

We further studied if hNPCs, alone, or encapsulated in bulk or granular HA hydrogels affected the proliferation of hPBMCs after co-culture in vitro for 5 d. Neither hNPCs encapsulated in bulk (19.0% ± 9.92%, *p* = 0.3382) nor granular HA hydrogel (25.1% ± 20.6%, *p* = 0.7121) resulted in any significant change in the proportion of CFSE^low^ population out of all CD3^+^ T cells compared to the hPBMC-only group (13.2% ± 7.69%, Figure 5b, *p* > 0.05). However, hNPCs in cell suspension (without TCA, Figure 5b,c) presented higher proportion of CFSE^low^ cells out of all CD3^+^ cells (16.3% ± 9.80%, *p* = 0.0136), as well as out of all CD3^−^ cells (18.2% ± 9.70%, *p* = 0.0182), when compared to the hPBMC-only group (13.2% ± 7.69% as in Figure 5b; 4.36% ± 4.20% as in Figure 5c).

### 3.7. hNPCs in Cell Suspension or Encapsulated in Bulk or Granular HA Hydrogel with TCA

We finally co-cultured hPBMCs with hNPCs either as a cell suspension or encapsulated in bulk or granular HA hydrogel with TCA. No significant change in the proportions of CFSE^low^ cells of all CD3^+^ hPBMCs was observed compared to the TCA-only group (control) (Figure 5d, *p* > 0.05). However, hNPCs, when added as a cell suspension, led to significantly larger proportion of CFSE^low^ cells out of the CD3^−^ subpopulation (49.0% ± 23.1%, *p* = 0.0058) compared to the TCA-only group (27.7% ± 15.2%, Figure 5e), while bulk or granular HA hydrogel with hNPCs encapsulated did not lead to similar effects (*p* > 0.05, Figure 5e).

### 3.8. Spidroin Preparations Still Contained Endotoxins

In order to investigate if endotoxins deriving from the bacteria used for recombinant spidroin production were still present, we performed a limulus amebocyte lysate (LAL) test on the soluble silk proteins. The results show that preparations of both spidroins contained endotoxins and that the VN–NT2RepCT solution had an approximate content of 380 EU/mg (Figure S4). Assuming that 10 EU corresponds to 1 ng endotoxin, the silk solutions added to the cultures contained around 38 EU endotoxin or 3.8 ng endotoxin.

## 4. Discussion

We set out to study the human biocompatibility of two potent but different biomaterials of interest for SCI repair, recombinant spidroins or HA hydrogels. We cultured hPBMCs with the biomaterials to mimic human blood–biomaterial interaction. We also co-cultured hPBMCs with allogeneic spinal cord-derived NPCs in the presence of these biomaterials of different forms. The aim was to mimic an allogeneic host (hPBMC)–donor (hNPC) cellular interaction with biomaterials as scaffolds in a composite experimental intervention. We monitored the human immune cell viability and proliferative response as well as evaluated the different immune cell phenotypes using NucleoCounter and flow cytometry.

We reported a high and stable hPBMC viability in the co-cultures with the various biomaterial types up to at least 4 days in vitro. No significant changes in viability of the experimental groups were observed compared to the hPBMC-only group. Little is known so far concerning the human immune cell viability and their responses after exposure to NT2RepCT. However, previous in vivo studies in rodents with subcutaneous implantation of the related 4RepCT variant of recombinant spider silk protein resulted in no observed systemic reaction and only minor local infiltration of macrophages and multinucleated cells 7 days postimplantation [27]. Hydrogel biomaterials, on the other hand, have been more widely studied as scaffold for different cells, for example, mesenchymal stem cells [54] or dendritic cells [55], with uncompromised viability. We did not expect to see changes in hPBMC viability under the present concentrations and conditions since the biomaterials evaluated in this study have been assessed in this regard previously. However, endotoxin contamination from gram negative bacteria is a common problem with recombinant proteins [56].

Human PBMCs contain approximately 70–90% lymphocytes (including T cells, B cells and natural killer (NK) cells), ~10% monocytes, 1–2% dendritic cells and small numbers of basophils, as described by Donaldson et al. [57]. The analysis of short-term responses by different hPBMC subpopulations revealed that CD4^+^ T cells, B cells and NK cells all became activated after co-culture with VN–NT2RepCT. In contrast, we did not see any significant activation after exposure to the HA hydrogels after this 15–18 h exposure. When co-culturing hPBMCs with recombinant spidroins in vitro, we did not observe a CD8^+^ T cell activation within this 15–18 h time span of biomaterial exposure. CD69 is a membrane-bound type II C-lectin receptor that is a classical early marker of lymphocyte activation [58]. CD69 expression is readily upregulated on most leukocytes as early as 2–3 h after stimulation. A number of different stimuli can drive expression of CD69, including engagement of the T cell receptors on T cells; stimulation of NK cells and T cells with cytokines such as IFN-a, IL-12 and IL-15; or LPS-mediated activation of B cells. As such, upregulation of CD69 can be used as a general marker of immune cell activation. However, we did not determine the detailed pathways of activation for the different immune cell subtypes after co-culture with artificial spidroins. One possible mechanism that could drive spidroin-induced immune activation could be low levels of LPS still remaining from the production, since the spidroin applied was produced in *E. coli*.

We also observed within 15–18 h an activation of innate NK cells as well as adaptive immune helper T and B cells but most probably not CD8^+^ T cells. With longer time exposure, a CD8^+^ T cell response would be expected toward foreign immunogenic materials as part of a foreign body response, as reviewed by Adusei et al. [59]. In the present study, the TCA control resulted in a more than threefold increase in the proportion of activated (CD69^+^) CD8^+^ T cells and CD4^+^ T cells, so a potential stimulation could take place under the present co-culture conditions also by CD8^+^ as well as CD4^+^ T cell subpopulations.

To analyze more long-term in vitro responses by the human immune cells, we followed the degree of proliferation of CD3^+^ and CD3^−^ hPBMC populations up to 5 days. When hPBMCs were cultured with the soluble or film form of NT2RepCT and VN–NT2RepCT or bulk or granular HA hydrogel, no significant increase in the proportion of the CFSE^low^ populations (including the highly proliferated population) was observed among CD3^+^ T cells compared to the hPBMC-only control group. In contrast, the CD3^−^ subpopulation presented significantly higher proportions of the CFSE^low^ populations after exposure to the soluble form of both NT2RepCT and VN–NT2RepCT compared to the hPBMC-only control. Within the CD3^−^ populations, there are a number of different cell types, including the fast-responding innate NK cells as well as innate B cells. Foreign body responses involving CD3^−^ cell types are well known and have, for example, been reported to result in extrusion of cochlear implants [60]. Furthermore, hydrogel-coated coils in experimental rat aneurysms have led to both neutrophil and B- and T cell presence in the implant area [61]. No previous studies have been performed concerning CD3^−^ cell populations toward spidroins such as NT2RepCT and VN–NT2RepCT. These spidroins have unique properties of interest for regenerative medicine, which calls for further production process development and evaluation. The present in vitro method to evaluate human immune compatibility with novel biomaterial candidates showed feasibility with high efficiency and relatively low resources.

Previous studies have shown that the molecular weight of HA may play a role in an induction or suppression of host immune response, but if the endotoxin contamination is low, not even the low molecular weight HA will provoke inflammation in vitro [62]. The HA applied in the present study is commercialized with a molecular weight of about 160 kDa, which is within the range of HA reported to be involved in a wide variety of biological processes in the body [63]. Furthermore, hyaluronidase-mediated catabolism of high-molecular-weight-HA into lower weights ranging between 0.5 and 27 kDa has been reported to not induce an immune response [64].

In the present study, we applied different forms of biomaterials to elucidate (in addition to biomaterial types) whether soluble vs. film or bulk vs. granular form could result in different compatibility outcomes. By introducing different forms of scaffold structure, we also exposed the human PBMCs to different concentrations of foreign materials. Cell proliferation of CD3^−^ cells significantly increased after 5 d in vitro culture with soluble VN–NT2RepCT and NT2RepCT but not with spidroin film compared to the hPBMC-only group. The observation may be due to higher exposure of biomaterials to the hPBMCs, when both biomaterial and immune cells are in the soluble/cell suspension form, which maximized the material exposure compared to a film coating at the base of the culture well. However, it may also be due to the amount of recombinant spidroin protein applied in the initial solution when coating the wells with a spidroin film (15 μg/well), while, in the spidroin-soluble condition, the resulting total concentration was as high as 100 μg/well at the time of seeding. The actual degree of spidroin film dissolvement into the culture medium is not known, but the spidroin-soluble conditions contained more of the recombinant spidroins compared to that under the film conditions. This may pose a dose-dependent outcome in our study. It is worth noting that we have previously cultured equivalent hNPCs (as applied in this study) with the recombinant spidroins VN–NT2RepCT and NT2RepCT in the form of fibers (15–50 micrometers in diameter) and film with final spidroin protein concentrations in the wells within the same range as in the present study. Under these concentrations, both survival and migration of hNPCs on spider silk films and fibers have been confirmed in our laboratories (data not shown).

The effects of recombinant silk proteins observed in this study could be a result of contaminating endotoxins from the expression of host bacteria, which would trigger human immune cells. In line with this, we investigated the endotoxin content of the soluble protein solutions by a LAL assay and found that the amount of endotoxin in the silk solutions added to the cultures were about 3.8 ng, which is similar to the positive control in which 10 ng LPS was added per well. Thus, the cellular activation observed for samples including silk protein preparations are likely due to the presence of endotoxin. The endotoxin content of recombinant spidroins produced in *E. coli* can be reduced to acceptable levels by a purification protocol that involves cell washes and multiple chromatographic steps [65]. Another convenient method would be to make scaffolds of the silk proteins and autoclave these, as this can reduce the endotoxin levels by 10- to 20-fold [66]. Having a relatively simple in vitro method to evaluate human immune cell responses along with the refinement of the production as shown here, is adding significant value.

The two different biomaterials studied here hold potential as candidate scaffolds for neural cell therapeutic strategies in nervous system disorders including SCI, either as an added singular biomaterial or potentially as a combination of granular HA hydrogel and VN–NT2RepCT, with or without donor neural cells. Previously, a combination of recombinant spidroin and hydrogel has been tested by others [67,68,69].

As a proof of principle for their use as a cell carrier, as a next step, allogeneic hNPCs derived from developing spinal cord tissues were added to cultures with hPBMCs and biomaterials. The hNPCs (from dissociated neurospheres) were added either as cell suspensions or as encapsulated cells in HA hydrogels. After 15–18 h of co-culture, flow cytometry analysis revealed that the hNPCs, alone, did not significantly increase the proportion of activated CD69^+^ or 4-1BB^+^ cells in any lymphocyte subpopulation compared to the hPBMC-only control group.

However, when cell suspensions of hNPCs were co-cultured with VN–NT2RepCT film, the proportion of activated CD69^+^ NK cells was increased significantly compared to the hPBMC-only group. In contrast, the granular HA hydrogel encapsulated hNPCs did not cause a significant effect to any of the hPBMC subpopulations examined and presented similar levels of activation as that of the hPBMC-only control group.

Finally, we also evaluated if encapsulation of hNPCs in either the form of bulk or granular hydrogels could elicit a proliferative response. We performed co-cultures for 5 days. Neither hNPCs encapsulated in bulk nor granular HA hydrogels resulted in any significant change in the proportion of CFSE^low^ population in CD3^+^ T cells or CD3^−^ cells compared to the hPBMC-only group, with or without TCA present. However, when hNPCs in cell suspension without TCA and any biomaterial were cultured as an additional control group, a higher proportion of proliferating CD3^+^ as well as CD3^−^ cells compared to the hPBMC-only group was observed. This observation is surprising, since both we and others have previously reported low immunogenicity by human neural cells [48,70]. When the hNPCs were encapsulated in hydrogels, this observation was not made. This could suggest that (1) an immune-compatible biomaterial may provide advantages in hindering direct cell–cell contact, (2) a potential immune response was elicited by the added “donor” human neural cells or (3) the added hNPCs here result in a false positive response by, themselves, adding to the CD3^−^ cell population since hNPCs were not prelabeled. The free cell suspension of hNPCs were mixed and in direct contact with hPBMCs in the culture media (in contrast to encapsulation in the hydrogel group) and were most probably included in the gated CD3^−^ population in the flow cytometry analysis and thereby could seemingly increase the proportion of the CD3^−^ subpopulation. The fact that, under TCA, the CD3^−^ but not the CD3^+^ hPBMC subpopulation was significantly increased with hNPC suspension added suggests that this is, indeed, the likely explanation. However, it is also known that prolonged in vitro culture of hNPCs in neurosphere culture will increase their cell surface expression of human leukocyte antigen I and II as well as result in costimulatory molecule expression, albeit still by a low number of neural cells [50]. It is therefore hypothesized that there is a low but potential risk for donor hNPCs to trigger a naïve lymphocyte response. In line with this hypothesis, attempts of human neural transplantations have included at least transient immunosuppressive regimens to reduce any potential risks [71,72,73].

Various progenitor/stem cell populations have previously been reported to have immunomodulatory features [48,74]. However, in the presence of T cell activation as under the present conditions, we did not observe any signs of immunomodulation by either the hNPCs in cell suspension or hNPCs encapsulated in bulk or granular HA hydrogel regarding CD3^+^ cell proliferation.

In this study, five different hPBMC donors and three different hNPC donor populations were studied. A shortcoming of the study is that not every step in the analysis was performed for all the experimental groups with varying types and forms of biomaterials. Despite the limited availability and accessibility of clinically relevant human materials not allowing us to test every combination of biomaterial form for every experiment, we still believe we here demonstrated a feasible approach to test multiple combinations of the different biological cases mimicking host (hPBMCs) and foreign donor (hNPCs) cellular interactions under the presence of biomaterials compared to control groups.

To evaluate biocompatibility of novel and potent biomaterials, most often, studies have been applied in animal, in vitro and in vivo, models [75,76,77]. In addition, when evaluating rodent or human immune cellular reactions to biomaterials, focus has often been on early innate responses [57,78], such as that recently thoroughly studied by Krüger-Genge et al. by their combined approach to test the immunocompatibility of gelatin-based hydrogels, of interest for production of vascular grafts [79]. In addition to experimental in vivo studies, including subcutaneous implantation in mice, they exposed human whole blood to hydrogels short-term for 4 h with subsequent analysis of cytokine expression and detected the release of complement factor C5a from human plasma by ELISA. By these methods, they sufficiently evaluated the short-term response by innate immune cells such as monocytes neutrophils as well as the complement system.

However, as argued by Adusei et al. [59] immunoengineering of biomaterials is still a developing field, where the next generation of implants in regenerative therapies must have a greater focus on T cell responses and adaptive immunity. Since the adaptive immune system has a long-term memory and is continuously reeducated, that will significantly affect biocompatibility. Each biomaterial design parameter will thereby contribute to this resulting immune response. To develop potent biomaterials for clinical application in nervous system disorders, it is therefore of value to be able to test human compatibility early on in the development process.

## 5. Conclusions

To conclude, we found that recombinant silk proteins contaminated with endotoxins from the production host significantly triggered a human immune response in vitro, including a T-, B- and NK cell response, while HA hydrogels did not stimulate equivalent human immune cells to activation or proliferation. It is resource effective to, early on in the development of new biomaterials aimed for clinical application, also evaluate their human biocompatibility. By applying a clinically relevant in vitro modeling system to assess human immune compatibility, the production process can be modified in parallel with biomaterial product development. We present a relatively simple and resource efficient method to evaluate biomaterial–hPBMC compatibility, including adaptive T cell responses, in conjunction with allogeneic human neural donor cells. This method offers the opportunity to detect potential immunogenic characteristics of the biomaterial of interest so that the material development and the production line can be optimized to reduce the risk of a negative foreign body response.

## Supplementary Materials

The following are available online at https://www.mdpi.com/article/10.3390/cells10071713/s1, Figure S1: Proportion of viable human PBMCs after exposure to different concentration of soluble spidroins. Figure S2: Flow cytometry gating strategy for the activation assay. Figure S3: Flow cytometry gating strategy for the proliferation assay. Figure S4. Standard curve of limolus amebocyte lyste (LAL) test. Table S1: Antibodies applied in the activation assay.

## References

[1] Badhiwala J.H., Ahuja C.S., Fehlings M.G. (2018). Time is spine: A review of translational advances in spinal cord injury. J. Neurosurg. Spine.

[2] Biering-Sorensen F., Bickenbach J.E., El Masry W.S., Officer A., von Groote P.M. (2011). ISCoS-WHO collaboration. International Perspectives of Spinal Cord Injury (IPSCI) report. Spinal. Cord.

[3] Alizadeh A., Dyck S.M., Karimi-Abdolrezaee S. (2019). Traumatic Spinal Cord Injury: An Overview of Pathophysiology, Models and Acute Injury Mechanisms. Front. Neurol..

[4] Liu S., Xie Y.Y., Wang B. (2019). Role and prospects of regenerative biomaterials in the repair of spinal cord injury. Neural Regen. Res..

[5] Thompson R.E., Pardieck J., Smith L., Kenny P., Crawford L., Shoichet M., Sakiyama-Elbert S. (2018). Effect of hyaluronic acid hydrogels containing astrocyte-derived extracellular matrix and/or V2a interneurons on histologic outcomes following spinal cord injury. Biomaterials.

[6] Li L.-M., Han M., Jiang X.-C., Yin X.-Z., Chen F., Zhang T.-Y., Ren H., Zhang J.-W., Hou T.-J., Chen Z. (2017). Peptide-Tethered Hydrogel Scaffold Promotes Recovery from Spinal Cord Transection via Synergism with Mesenchymal Stem Cells. ACS Appl. Mater. Interfaces.

[7] Zaviskova K., Tukmachev D., Dubisova J., Vackova I., Hejcl A., Bystronova J., Pravda M., Scigalkova I., Sulakova R., Velebny V. (2018). Injectable hydroxyphenyl derivative of hyaluronic acid hydrogel modified with RGD as scaffold for spinal cord injury repair. J. Biomed. Mater. Res. Part A.

[8] Han S., Wang B., Jin W., Xiao Z., Li X., Ding W., Kapur M., Chen B., Yuan B., Zhu T. (2015). The linear-ordered collagen scaffold-BDNF complex significantly promotes functional recovery after completely transected spinal cord injury in canine. Biomaterials.

[9] Li X., Zhao Y., Cheng S., Han S., Shu M., Chen B., Chen X., Tang F., Wang N., Tu Y. (2017). Cetuximab modified collagen scaffold directs neurogenesis of injury-activated endogenous neural stem cells for acute spinal cord injury repair. Biomaterials.

[10] Koffler J., Zhu W., Qu X., Platoshyn O., Dulin J.N., Brock J., Graham L., Lu P., Sakamoto J., Marsala M. (2019). Biomimetic 3D-printed scaffolds for spinal cord injury repair. Nat. Med..

[11] Rooney G.E., Knight A.M., Madigan N.N., Gross L., Chen B., Giraldo C.V., Seo S., Nesbitt J.J., Dadsetan M., Yaszemski M.J. (2011). Sustained delivery of dibutyryl cyclic adenosine monophosphate to the transected spinal cord via oligo [(polyethylene glycol) fumarate] hydrogels. Tissue Eng. Part A.

[12] Kornfeld T., Nessler J., Helmer C., Hannemann R., Waldmann K.H., Peck C.T., Hoffmann P., Brandes G., Vogt P.M., Radtke C. (2021). Spider silk nerve graft promotes axonal regeneration on long distance nerve defect in a sheep model. Biomaterials.

[13] Radtke C., Allmeling C., Waldmann K.-H., Reimers K., Thies K., Schenk H.C., Hillmer A., Guggenheim M., Brandes G., Vogt P.M. (2011). Spider Silk Constructs Enhance Axonal Regeneration and Remyelination in Long Nerve Defects in Sheep. PLoS ONE.

[14] Hunt J.A., Chen R., van Veen T., Bryan N. (2014). Hydrogels for tissue engineering and regenerative medicine. J. Mater. Chem. B.

[15] Xue K., Wang X., Yong P.W., Young D.J., Wu Y.-L., Li Z., Loh X.J. (2019). Hydrogels as Emerging Materials for Translational Biomedicine. Adv. Ther..

[16] Gosline J.M., Guerette P.A., Ortlepp C.S., Savage K.N. (1999). The mechanical design of spider silks: From fibroin sequence to mechanical function. J. Exp. Biol..

[17] Salehi S., Koeck K., Scheibel T. (2020). Spider Silk for Tissue Engineering Applications. Molecules.

[18] Vollrath F., Barth P., Basedow A., Engstrom W., List H. (2002). Local tolerance to spider silks and protein polymers in vivo. In Vivo.

[19] Varone A., Knight D., Lesage S., Vollrath F., Rajnicek A.M., Huang W. (2017). The potential of Antheraea pernyi silk for spinal cord repair. Sci. Rep..

[20] Chung H., Kim T.Y., Lee S.Y. (2012). Recent advances in production of recombinant spider silk proteins. Curr. Opin. Biotechnol..

[21] Rising A., Johansson J. (2015). Toward spinning artificial spider silk. Nat. Chem. Biol..

[22] Andersson M., Jia Q., Abella A., Lee X.-Y., Landreh M., Purhonen P., Hebert H., Tenje M., Robinson C.V., Meng Q. (2017). Biomimetic spinning of artificial spider silk from a chimeric minispidroin. Nat. Chem. Biol..

[23] Lewicka M., Hermanson O., Rising A.U. (2012). Recombinant spider silk matrices for neural stem cell cultures. Biomaterials.

[24] Widhe M., Bysell H., Nystedt S., Schenning I., Malmsten M., Johansson J., Rising A., Hedhammar M. (2010). Recombinant spider silk as matrices for cell culture. Biomaterials.

[25] Wu S., Johansson J., Hovatta O., Rising A. (2015). Efficient passage of human pluripotent stem cells on spider silk matrices under xeno-free conditions. Cell. Mol. Life Sci..

[26] Wu S., Johansson J., Damdimopoulou P., Shahsavani M., Falk A., Hovatta O., Rising A. (2014). Spider silk for xeno-free long-term self-renewal and differentiation of human pluripotent stem cells. Biomaterials.

[27] Fredriksson C., Hedhammar M., Feinstein R., Nordling K., Kratz G., Johansson J., Huss F., Rising A. (2009). Tissue Response to Subcutaneously Implanted Recombinant Spider Silk: An in Vivo Study. Materials.

[28] Vecino E., Kwok J.C.F. (2016). The Extracellular Matrix in the Nervous System: The Good and the Bad Aspects. Composition and Function of the Extracellular Matrix in the Human Body.

[29] Khaing Z.Z., Milman B.D., Vanscoy J.E., Seidlits S.K., Grill R.J., Schmidt C.E. (2011). High molecular weight hyaluronic acid limits astrocyte activation and scar formation after spinal cord injury. J. Neural Eng..

[30] Ahmed E.M. (2015). Hydrogel: Preparation, characterization, and applications: A review. J. Adv. Res..

[31] Peppas N.A., Bures P., Leobandung W., Ichikawa H. (2000). Hydrogels in pharmaceutical formulations. Eur. J. Pharm. Biopharm..

[32] Assuncao-Silva R.C., Gomes E.D., Sousa N., Silva N.A., Salgado A.J. (2015). Hydrogels and Cell Based Therapies in Spinal Cord Injury Regeneration. Stem Cells Int..

[33] Schizas N., Rojas R., Kootala S., Andersson B., Pettersson J., Hilborn J., Hailer N.P. (2014). Hyaluronic acid-based hydrogel enhances neuronal survival in spinal cord slice cultures from postnatal mice. J. Biomater. Appl..

[34] Madl C.M., LeSavage B.L., Dewi R.E., Lampe K.J., Heilshorn S.C. (2019). Matrix Remodeling Enhances the Differentiation Capacity of Neural Progenitor Cells in 3D Hydrogels. Adv. Sci..

[35] Madl C.M., LeSavage B.L., Dewi R.E., Dinh C.B., Stowers R.S., Khariton M., Lampe K.J., Nguyen D., Chaudhuri O., Enejder A. (2017). Maintenance of neural progenitor cell stemness in 3D hydrogels requires matrix remodelling. Nat. Mater..

[36] Hsu C.-C., George J.H., Waller S., Besnard C., Nagel D., Hill E., Coleman M.D., Korsunsky A.M., Cui Z., Ye H. Increased Connectivity of hiPSC-derived Neural Networks in Multiphase of Granular Hydrogel Scaffolds. Bioact. Mater..

[37] Riley L., Schirmer L., Segura T. (2018). Granular hydrogels: Emergent properties of jammed hydrogel microparticles and their applications in tissue repair and regeneration. Curr. Opin. Biotechnol..

[38] George J., Hsu C.C., Nguyen L.T.B., Ye H., Cui Z. (2019). Neural tissue engineering with structured hydrogels in CNS models and therapies. Biotechnol. Adv..

[39] Bergman K., Engstrand T., Hilborn J., Ossipov D., Piskounova S., Bowden T. (2009). Injectable cell-free template for bone-tissue formation. J. Biomed. Mater. Res. Part A.

[40] Siegelman M.H., DeGrendele H.C., Estess P. (1999). Activation and interaction of CD44 and hyaluronan in immunological systems. J. Leukoc. Biol..

[41] Kajahn J., Franz S., Rueckert E., Forstreuter I., Hintze V., Moeller S., Simon J.C. (2012). Artificial extracellular matrices composed of collagen I and high sulfated hyaluronan modulate monocyte to macrophage differentiation under conditions of sterile inflammation. Biomatter.

[42] Nakamura K., Yokohama S., Yoneda M., Okamoto S., Tamaki Y., Ito T., Okada M., Aso K., Makino I. (2004). High, but not low, molecular weight hyaluronan prevents T-cell-mediated liver injury by reducing proinflammatory cytokines in mice. J. Gastroenterol..

[43] Kwon M.Y., Wang C., Galarraga J.H., Pure E., Han L., Burdick J.A. (2019). Influence of hyaluronic acid modification on CD44 binding towards the design of hydrogel biomaterials. Biomaterials.

[44] Luttikhuizen D.T., Harmsen M.C., Van Luyn M.J. (2006). Cellular and molecular dynamics in the foreign body reaction. Tissue Eng..

[45] Jones J.A., Chang D.T., Meyerson H., Colton E., Kwon I.K., Matsuda T., Anderson J.M. (2007). Proteomic analysis and quantification of cytokines and chemokines from biomaterial surface-adherent macrophages and foreign body giant cells. J. Biomed. Mater. Res. Part A.

[46] Andorko J.I., Jewell C.M. (2017). Designing biomaterials with immunomodulatory properties for tissue engineering and regenerative medicine. Bioeng. Transl. Med..

[47] O’Shea T.M., Wollenberg A.L., Kim J.H., Ao Y., Deming T.J., Sofroniew M.V. (2020). Foreign body responses in mouse central nervous system mimic natural wound responses and alter biomaterial functions. Nat. Commun..

[48] Odeberg J., Piao J.H., Samuelsson E.B., Falci S., Akesson E. (2005). Low immunogenicity of in vitro-expanded human neural cells despite high MHC expression. J. Neuroimmunol..

[49] Piao J.H., Odeberg J., Samuelsson E.B., Kjaeldgaard A., Falci S., Seiger A., Sundstrom E., Akesson E. (2006). Cellular composition of long-term human spinal cord- and forebrain-derived neurosphere cultures. J. Neurosci. Res..

[50] Liu J., Gotherstrom C., Forsberg M., Samuelsson E.B., Wu J., Calzarossa C., Hovatta O., Sundstrom E., Akesson E. (2013). Human neural stem/progenitor cells derived from embryonic stem cells and fetal nervous system present differences in immunogenicity and immunomodulatory potentials in vitro. Stem Cell Res..

[51] Hsu C.-C., George J.H., Waller S., Besnard C., Korsunsky A.M., Ye H., Cui Z. Culture of Human Induced Pluripotent Stem Cell-Derived Neurons in Multi-phase Granular Hydrogels. Proceedings of the Biomaterials for Cell and Drug Delivery 2019.

[52] Sancho D., Gómez M., Sánchez-Madrid F. (2005). CD69 is an immunoregulatory molecule induced following activation. Trends Immunol..

[53] Vinay D.S., Kwon B.S. (1998). Role of 4-1BB in immune responses. Semin. Immunol..

[54] Aleksander-Konert E., Paduszyński P., Zajdel A., Dzierżewicz Z., Wilczok A. (2016). In vitro chondrogenesis of Wharton’s jelly mesenchymal stem cells in hyaluronic acid-based hydrogels. Cell. Mol. Biol. Lett..

[55] Groell F., Kalia Y.N., Jordan O., Borchard G. (2018). Hydrogels in three-dimensional dendritic cell (MUTZ-3) culture as a scaffold to mimic human immuno competent subcutaneous tissue. Int. J. Pharm..

[56] Mamat U., Wilke K., Bramhill D., Schromm A.B., Lindner B., Kohl T.A., Corchero J.L., Villaverde A., Schaffer L., Head S.R. (2015). Detoxifying Escherichia coli for endotoxin-free production of recombinant proteins. Microb. Cell Factories.

[57] Donaldson A.R., Tanase C.E., Awuah D., Vasanthi Bathrinarayanan P., Hall L., Nikkhah M., Khademhosseini A., Rose F., Alexander C., Ghaemmaghami A.M. (2018). Photocrosslinkable Gelatin Hydrogels Modulate the Production of the Major Pro-inflammatory Cytokine, TNF-α, by Human Mononuclear Cells. Front. Bioeng. Biotechnol..

[58] Cibrian D., Sanchez-Madrid F. (2017). CD69: From activation marker to metabolic gatekeeper. Eur. J. Immunol..

[59] Adusei K.M., Ngo T.B., Sadtler K. (2021). T Lymphocytes as Critical Mediators in Tissue Regeneration, Fibrosis, and the Foreign Body Response. Acta Biomater..

[60] Nadol J.B., O’Malley J.T., Burgess B.J., Galler D. (2014). Cellular immunologic responses to cochlear implantation in the human. Hear. Res..

[61] Zhang C., Chaudhary N., Gemmete J.J., Thompson B.G., Xi G., Pandey A.S. (2014). Reactive tissue proliferation and damage of elastic lamina caused by hydrogel coated coils in experimental rat aneurysms. J. Neurointerv. Surg..

[62] Baeva L.F., Lyle D.B., Rios M., Langone J.J., Lightfoote M.M. (2014). Different molecular weight hyaluronic acid effects on human macrophage interleukin 1β production. J. Biomed. Mater. Res. Part A.

[63] Snetkov P., Zakharova K., Morozkina S., Olekhnovich R., Uspenskaya M. (2020). Hyaluronic Acid: The Influence of Molecular Weight on Structural, Physical, Physico-Chemical, and Degradable Properties of Biopolymer. Polymers.

[64] Cyphert J.M., Trempus C.S., Garantziotis S. (2015). Size Matters: Molecular Weight Specificity of Hyaluronan Effects in Cell Biology. Int. J. Cell Biol..

[65] Hedhammar M., Bramfeldt H., Baris T., Widhe M., Askarieh G., Nordling K., Aulock S., Johansson J. (2010). Sterilized recombinant spider silk fibers of low pyrogenicity. Biomacromolecules.

[66] Decker R.E., Harris T.I., Memmott D.R., Peterson C.J., Lewis R.V., Jones J.A. (2018). Method for the Destruction of Endotoxin in Synthetic Spider Silk Proteins. Sci. Rep..

[67] Schacht K., Scheibel T. (2011). Controlled Hydrogel Formation of a Recombinant Spider Silk Protein. Biomacromolecules.

[68] Osama I., Gorenkova N., McKittrick C.M., Wongpinyochit T., Goudie A., Seib F.P., Carswell H.V.O. (2018). In vitro studies on space-conforming self-assembling silk hydrogels as a mesenchymal stem cell-support matrix suitable for minimally invasive brain application. Sci. Rep..

[69] Hopkins A.M., De Laporte L., Tortelli F., Spedden E., Staii C., Atherton T.J., Hubbell J.A., Kaplan D.L. (2013). Silk Hydrogels as Soft Substrates for Neural Tissue Engineering. Adv. Funct. Mater..

[70] Ozaki M., Iwanami A., Nagoshi N., Kohyama J., Itakura G., Iwai H., Nishimura S., Nishiyama Y., Kawabata S., Sugai K. (2017). Evaluation of the immunogenicity of human iPS cell-derived neural stem/progenitor cells in vitro. Stem Cell Res..

[71] Akesson E., Wolmer-Solberg N., Cederarv M., Falci S., Odeberg J. (2009). Human neural stem cells and astrocytes, but not neurons, suppress an allogeneic lymphocyte response. Stem Cell Res..

[72] Ubiali F., Nava S., Nessi V., Frigerio S., Parati E., Bernasconi P., Mantegazza R., Baggi F. (2007). Allorecognition of human neural stem cells by peripheral blood lymphocytes despite low expression of MHC molecules: Role of TGF-beta in modulating proliferation. Int. Immunol..

[73] Ekblad-Nordberg Å., Walther-Jallow L., Westgren M., Götherström C. (2020). Prenatal stem cell therapy for inherited diseases: Past, present, and future treatment strategies. Stem Cells Transl. Med..

[74] Urdzikova L.M., Ruzicka J., LaBagnara M., Karova K., Kubinova S., Jirakova K., Murali R., Sykova E., Jhanwar-Uniyal M., Jendelova P. (2014). Human mesenchymal stem cells modulate inflammatory cytokines after spinal cord injury in rat. Int. J. Mol. Sci..

[75] Sirova M., Vlierberghe S.V., Matyasova V., Rossmann P., Schacht E., Dubruel P., Rihova B. (2014). Immunocompatibility evaluation of hydrogel-coated polyimide implants for applications in regenerative medicine. J. Biomed. Mater. Res. Part A.

[76] Kłodzińska S.N., Pletzer D., Rahanjam N., Rades T., Hancock R.E.W., Nielsen H.M. (2019). Hyaluronic acid-based nanogels improve in vivo compatibility of the anti-biofilm peptide DJK-5. Nanomed. Nanotechnol. Biol. Med..

[77] Kampleitner C., Obi J., Vassilev N., Epstein M.M., Hoffmann O. (2018). Biological Compatibility Profile on Biomaterials for Bone Regeneration. J. Vis. Exp..

[78] Arthe R., Arivuoli D., Ravi V. (2020). Preparation and characterization of bioactive silk fibroin/paramylon blend films for chronic wound healing. Int. J. Biol. Macromol..

[79] Krüger-Genge A., Tondera C., Hauser S., Braune S., Görs J., Roch T., Klopfleisch R., Neffe A.T., Lendlein A., Pietzsch J. (2021). Immunocompatibility and non-thrombogenicity of gelatin-based hydrogels. Clin. Hemorheol. Microcirc..

