# Supplementary material for: In Vitro Study of Human Immune Responses to Hyaluronic Acid Hydrogels, Recombinant Spidroins and Human Neural Progenitor Cells of Relevance to Spinal Cord Injury Repair
Source: Cells. 2021 Jul 6;10(7):1713. doi: 10.3390/cells10071713 (PMC8303367; doi:10.3390/cells10071713)
Supplement: Supplementary file 1 [file cells-10-01713-s001.zip › Cells-1273154-Lin et al., Cells-Figure S1,S2,S3,S4 revised.pdf]

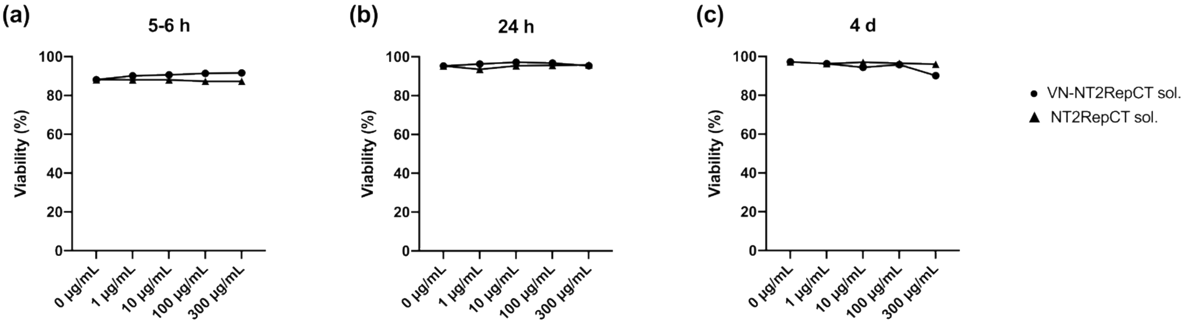

Figure S1. Proportion of viable human PBMCs after exposure to different concentration of soluble spidroins. Prior to the proliferation assay, different concentrations of soluble spidroins of either NT2RepCT or VN-NT2RepCT type (1 µg/mL, 10 µg/mL, 100 µg/mL, 300 µg/mL) were co-cultured with hPBMCs from one donor for 5-6 h (a), 24 h (b), or 4 d (c) after which the hPBMC viability was analysed by NucleoCounter. The proportion of viable hPBMCs were in the following ranges after 5-6 h (87.3 - 91.6%), 24 h (93.5 - 97.2%), 4 d (90.1- 97.2%), similar to the control with no added spidroin protein.

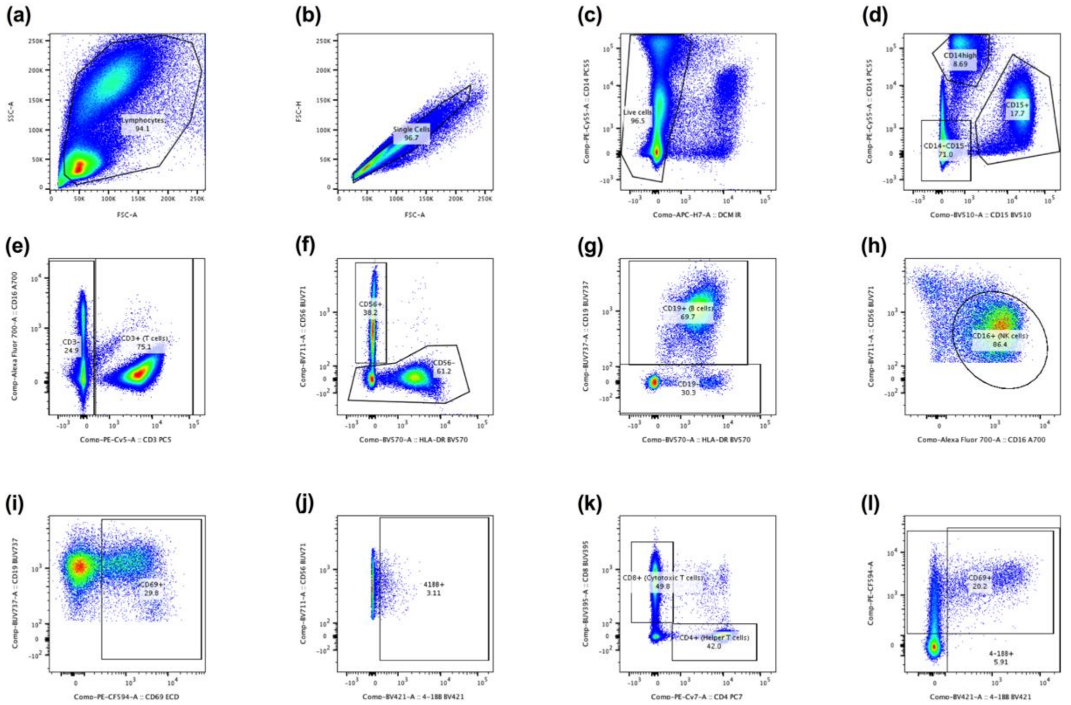

Figure S2. Flow cytometry gating strategy for the activation assay.

(a) Total lymphocytes were gated on a forward scatter (FSC)/side scatter (SSC) plot. (b) Single cells were gated using forward scatter area and height. (c) Live/Dead fixable near-IR stain kit was used to label live cells (fluorescence negative cells in the left). (d) Live cells were then further gated for CD14 and CD15, where CD14$^-$ CD15$^-$ population (lower left) were gated for the subsets of interest, namely B cells (CD3$^-$CD56$^-$CD19$^+$, e→f→g) , NK cells (CD3$^-$CD56$^+$CD16$^+$, e→f→h), helper T cells (CD3$^+$CD4$^+$, e→k), cytotoxic T cells (CD3$^+$CD8$^+$, e→k). We then further gated B cells, NK cells, helper T cells and cytotoxic T cells for CD69$^+$ and 4-1BB$^+$ subpopulation (i, j, l). Data was analyzed using FlowJo software, and population frequencies expressed as percent of the parent populations.

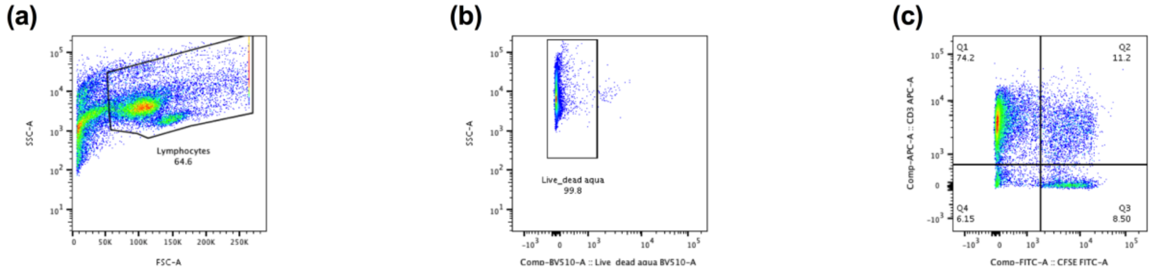

Figure S3. Flow cytometry gating strategy for the proliferation assay.

(a) Total lymphocytes were gated on a forward scatter (FSC)/side scatter (SSC) plot. (b) Live cells were gated using side scatter area and live/dead Aqua negative cells (left). (c) The live cells were then further gated for the proliferated $CD3^+$ cells ($CFSE^{low}$ $CD3^+$, upper left) and proliferated $CD3^-$ cells ($CFSE^{low}$ $CD3^-$, lower left). Data was analyzed using FlowJo software and the population frequencies represent percentage of the $CD3^+$ and $CD3^-$ parent population.

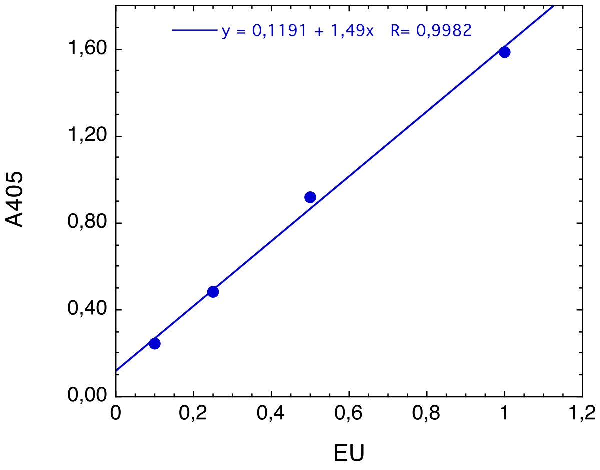

Figure S4. Standard curve of *limolus amebocyte lyste* (LAL) test.

The VN-NT2RepCT solution was diluted in endotoxin free water, 10, 100 and 1000 times and remeasured. The two first dilutions gave maximum absorbance (3.5), while the 1000 times dilution sample gave an absorbance of 1.58 which corresponds to approximately 1 EU/mL (1 EU per 2.6 µg VN-NT2RepCT) or approximately 380 EU per mg VN-NT2RepCT. 3 mg/mL solution of NT2RepCT gave an absorbance that was > 3.5, that is >>1 EU per mL 2.6 mg/mL solution of VN-NT2RepCT gave an absorbance that was > 3.5, that is >>1 EU per mL.
