# Supplementary material for: In Vitro Study of Human Immune Responses to Hyaluronic Acid Hydrogels, Recombinant Spidroins and Human Neural Progenitor Cells of Relevance to Spinal Cord Injury Repair
Source: Cells. 2021 Jul 6;10(7):1713. doi: 10.3390/cells10071713 (PMC8303367; doi:10.3390/cells10071713)
Supplement: Supplementary file 1 [file cells-10-01713-s001.zip › Table.pdf]

Table S1. Antibodies applied in the activation assay.

**Antibody-Flurochrome**	**Clone**	**Manufacture**
CD11c-FITC	KB90	Dako Cytomation
CD86-APC	2331 (FUN-1)	BD Biosciences
CD16-Alexa700	3G2	BD Biosciences
Live/Dead	Live/Dead Fixable near-IR Dead cell kit	Invitrogen
4-1BB-BV421	4B4-1	BioLegend
CD15-BV510	W6D3	BioLegend
HLA-DR-BV570	L243	BioLegend
CD56-BV711	HCD56	BioLegend
CD4-BV750	SK3	BioLegend
CD69-ECD	TP1.55.3	Coulter
CD3-PE-Cy5	UCHT1	BD Biosciences
CD14-PE-Cy5.5	RMO52	Beckman Coulter
CD8-BUV395	RPA-T8	BD Biosciences
CD19-BUV737	SJ25C1	BD Biosciences
